# Population Mass Balance Model for Precipitation with Turbidity Measurements

**DOI:** 10.1021/acsomega.3c10516

**Published:** 2024-03-05

**Authors:** Paszkál Papp, Ágota Tóth, Dezső Horváth

**Affiliations:** † Department of Physical Chemistry and Materials Science, University of Szeged, Szeged 6720, Hungary; ‡ Department of Applied and Environmental Chemistry, University of Szeged, Szeged 6720, Hungary

## Abstract

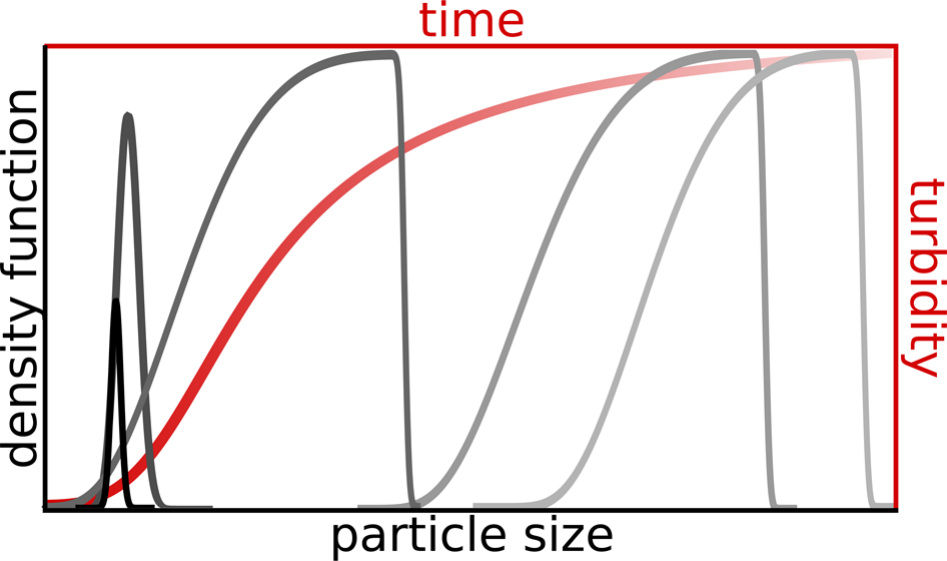

The discretized population balance theory has been proven to be a useful method to simulate systems in which solid particles are present. In this work, we introduce a new approach to model precipitation reactions based on the temporal evolution of product concentration, from which particle size distribution, its dynamics, and the specific interfacial energies can be obtained. For a reference study, the previously investigated calcium oxalate precipitation was selected, where the reaction was followed via turbidity measurement. From the obtained particle size distribution, we can show that at low supersaturation, growth is the dominant process, while at higher supersaturation, nucleation is the dominant process. Moreover, the temporal change of the distribution curve has allowed us to split the precipitation into a nucleation, a growth-driven intermediate, and a saturation regime. Furthermore, the comparison between the experimental and calculated results has proved that the method is suitable for predicting particle size distributions and specific interfacial energies.

## Introduction

The computational modeling of heterogeneous systems, in which solid or liquid particles are dispersed in a fluid medium, can be a challenging task, therefore it has been in the focus of extensive scientific investigation lately.^1−3^ Precipitation can generate such systems; their simulation is especially difficult because the number and sizes of the elements are not stationary. There are processes that increase or decrease the number of particles, such as nucleation, redissolution, or agglomeration, to name a few. Moreover, their sizes can also change dynamically via growth or breakage.^4^ Because the system is heterogeneous, it cannot be described by using solely the classical state variables, since the characteristic properties of the dispersed elements, such as their size, habit, structure, or configuration in continuous phase, have to be taken into account as well.^5^ In order to simulate systems coupled with precipitation reactions, the size, the composition, and the trajectory of each particle at every time instance must be calculated.^6,7^ However, in some cases, for example, in industrial crystallization processes, the number of particles becomes vast to follow them individually.^8^ For these cases, the discretized population balance (DPB) model was developed by Hulburt and Katz.^9^ In their work, instead of treating particles separately, a density function is used to describe the particle size distribution (PSD) and the evolution of the precipitate. Their formula has been applied in various fields of research, for example, in the modeling of bacteria division,^10^ in electrochemistry,^11^ or in polymer chemistry.^12^

In many studies concerning DPB modeling, the measured PSD is used to fit the parameters appearing in the density function.^13,14^ In this work, we present a method to predict PSDs using temporal precipitate concentration curves. The evolution of product concentration in the case of precipitation can be followed with various experimental techniques, for example, by measuring conductance,^15^ dynamic light scattering,^16^ or turbidity. The latter can be conveniently monitored utilizing a photometer^17−20^ or a high-speed camera.^21^ As a case study, a previously investigated calcium oxalate formation^15^ is selected, from which the temporal evolution of turbidity for various initial reactant concentrations and the scanning electron microscopy (SEM) images of the products are available. By utilizing our new method, we can predict the PSD to provide information on the precipitate size domain.

## Modeling

### Governing Equations

Isothermal crystallization is considered where the formation of solid phase is separated into two subprocesses: nucleation, which depends solely on supersaturation *S*, and particle growth, which is also affected by the surface area of the particles (*A*_p_) according to
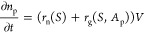
1where *n*_p_ is the amount of substance of the product, *V* is the volume of the solution, and *r*_n_ and *r*_g_ are the rates of nucleation and growth, respectively. Due to the large number of particles, their sizes cannot be treated individually; therefore, DPB theory has been applied. The PSD function *f*(*L*) is defined as
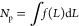
2which is an unnormalized density function, where *N*_p_ is the number of particles in the system and *L* is the particle size. In this model the particles are chosen to be spherical; thus, *L* is equal to the diameter of the sphere. By taking into account the definition of the PSD function eq 2 and the assumption that the particles are spherical, the total surface area (*A*_p_) and volume (*V*_p_) of the solid phase can be given as
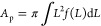
3
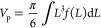
4

Since the PSD function is used to describe the number and size distribution of the solid particles, its temporal evolution depends on the formation of the solid phase; therefore, it can also be divided into two subprocesses. Thus, the temporal change of the PSD function can be expressed as
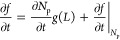
5where the first term corresponds to the nucleation and the second to the growth, and *g*(*L*) is a normal distribution function. In our model, solely homogeneous nucleation is considered; hence, the temporal change of the particles is proportional to the rate of nucleation, i.e.,
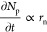
6We assume that particles do not form with a uniform size, instead, nucleation leads to a certain size distribution^22^*g*(*L*), which we approximate with a normal distribution function
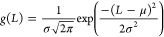
7where μ and σ is the mean size and its standard deviation, respectively. The mean is set to be μ = 1 μm, matching the upper limit of the colloidal size range.^23^ In order to introduce stochasticity into the model, the initial distribution function has to be sufficiently wide; therefore, for standard deviation, σ = 0.1 μm is chosen arbitrarily. By the usage of such μ and σ parameter values, the *g*(*L*) initial normal distribution function is in the positive particle size domain, and the probability of negative diameters is negligible.

Since the evolution of the particle diameter depends only on the precipitation process, the temporal change in the *i*-th particle size is proportional to its growth rate
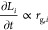
8

As a case study, the precipitation of calcium oxalate in a cuvette is investigated. The reaction can be described with equation
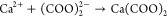
9In our model, the particles nucleate via primary nucleation. Due to the short examination times, secondary nucleation, agglomeration, and breakage are assumed to be negligible. The rate of nucleation according to the classical nucleation theory^24^ can be given as
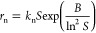
10where *k*_n_ is the nucleation rate coefficient and *S* is the supersaturation defined as
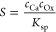
11where *c*_Ca_ and *c*_Ox_ are the concentration of the reactant calcium and oxalate ions, respectively, and *K*_sp_ is the solubility product. The thermodynamic parameter *B* is defined as
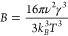
12where ν is the molecular volume of the solid particle, γ is the specific interfacial energy, *k*_*B*_ is the Boltzmann constant, and *T* is the absolute temperature.

The growth of the solid particles depends not only on supersaturation but also on the surface area of the particles present in the given volume.^7^ Thus, the growth rate of the solid precipitate (*r*_g_ = ∑_*i*_*r*_g,*i*_) can be expressed as
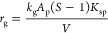
13where *k*_g_ is the growth rate coefficient.

By taking into account that the particles are spherical, the amount of substance formed due to nucleation can be given as
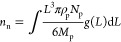
14where ρ_p_ and *M* are the density and the molar mass of the solid particle, respectively. By rearranging eq 14, the temporal change of the particle number can be given as
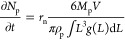
15

The temporal change in the amount of substance formed due to the growth of the *i*-th spherical particle (*n*_g,*i*_) can be expressed as
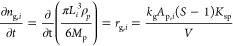
16where *A*_p,*i*_ is the surface of the *i*-th crystal, from which, after rearrangements, the temporal change in the particle volume can be given as
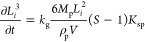
17Rearranging the equation and performing the derivation, we find that the temporal diameter growth of the *i*-th particle is independent of its own size
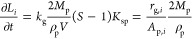
18thus the growth of the particles can be given using the growth rate *r*_g_ and the total surface area of the precipitate
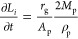
19where *A*_p_ is obtained by numerical integration according to eq 3.

The second term in eq 5, which governs the crystal size dynamics, is formulated by taking the size dependence of the *f*(*L*) function and applying the chain rule
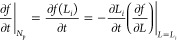
20The negative sign indicates that the function *f*(*L*) is shifted toward the larger particle sizes as the particles grow.

### Numerical Methods

**Figure 1:**
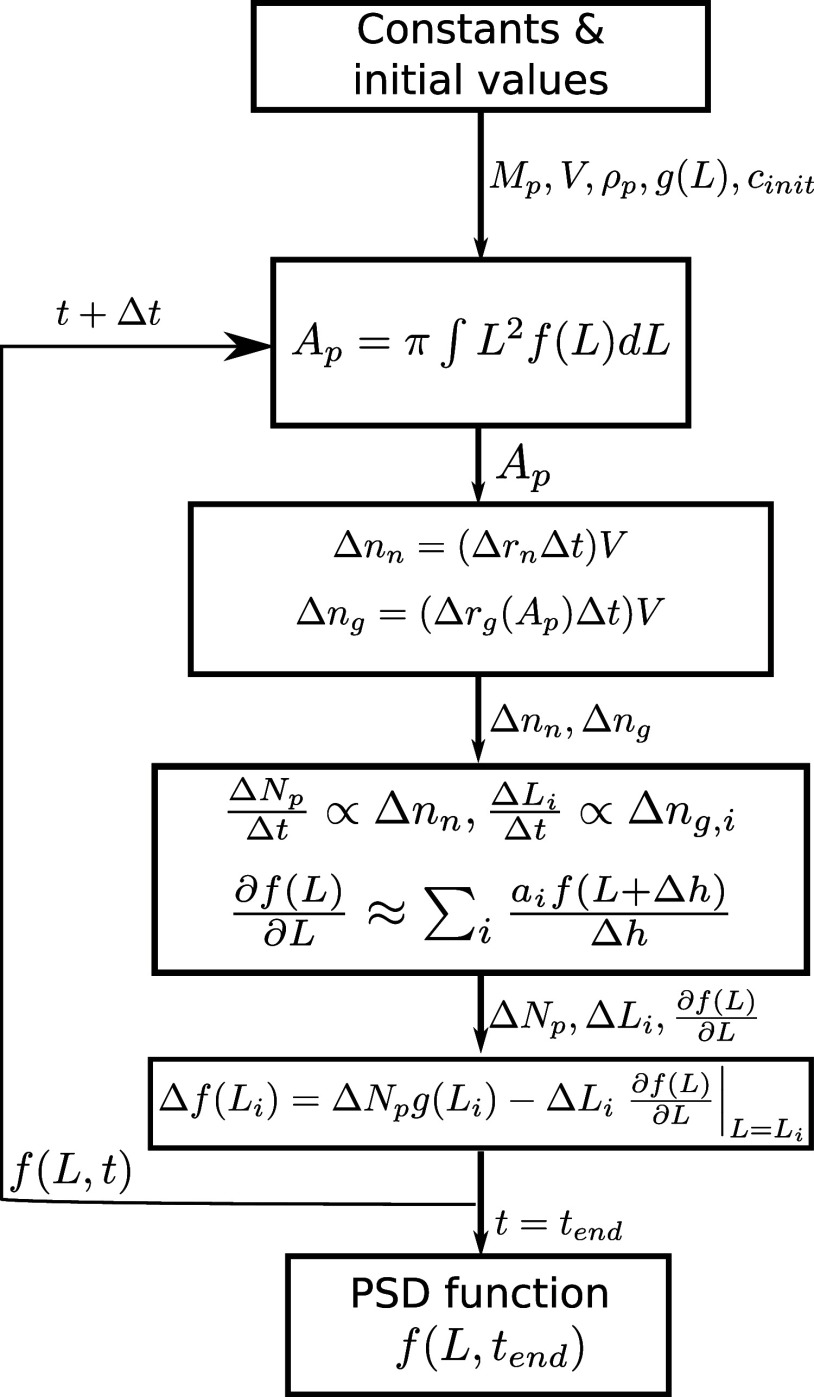
Algorithm of the steps applied in the numerical scheme.

The numerical solution of eq 5 is based on an explicit Euler scheme, with the algorithm presented in Figure 1. From the initial parameters and constants, the surface area of particles (*A*_p_) is determined in order to obtain the change in the amount of substance due to nucleation (Δ*n*_n_) and growth (Δ*n*_g_). This allows the update of reactant concentration according to
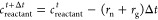
21where *c*_reactant_^*t*^ stands for the concentration of calcium ion and oxalate ion. The changes in particle number and size according to eqs 15 and 19 are then obtained. The ∂*f*(*L*)/∂*L* term is calculated, followed by an evaluation of the updated PSD function. The procedure is iterated until the end time (*t*_end_) is reached.

The PSD function is discretized on 20,000 points with a spacing of 0.005 μm. The trapezoidal rule is applied to calculate both the surface area eq 3 and the volume of the particles eq 4. The ∂*f*(*L*)/∂*L* term in eq 5 is approximated by the central finite difference formula with a sixth-order accuracy. The PSD function is smoothed with a three–point moving average method in every 200th iteration. The simulations with a time step of Δ*t* = 0.001 s are performed with an in-house solver written in C coupled with OpenMPI, enabling parallel computation, thus reducing the run time significantly.

**Figure 2:**
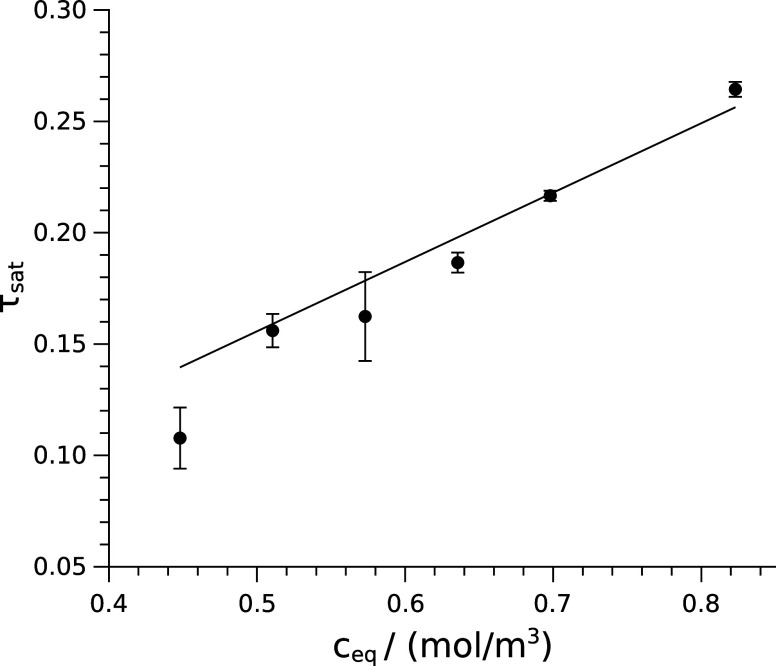
Calibration used to convert turbidity to the amount of product in the experimental data set.^15^

For a reference data set, the previously measured temporal turbidity evolution for Ca(COO)_2_ precipitation is used,^15^ where it was shown that the turbidity dependence on concentration follows a relationship analogous to the Lambert–Beer law at the experimental conditions. Hence, the turbidity can be converted to the amount of substance in a unit volume for the product. For calibration, the saturation turbidity values are plotted as a function of equilibrium composition (*c*_eq_), as shown in Figure 2. The amount of the product at equilibrium is obtained from , where . The slope of the calibration curve presented in Figure 2 is found to be (0.3114 ± 0.0045) m^3^/mol with zero intercept.

The physical constants used in the calculations are summarized in Table 1. The same temperature is set as in the experiments, and *V* is equal to the volume of solution in the cuvette. The kinetic reaction rate coefficient for nucleation (*k*_n_) and growth (*k*_g_) and the thermodynamic parameter (*B*) are unknown; they have been determined via optimization by using Monte Carlo and simplex methods with the relative tolerance of 10^–3^.

**Table 1:** Physical Quantities Used in Calculations

quantity	value	ref.
*T*	298 K	
*V*	4 cm^3^	
*K*_sp_	2.7 × 10^–3^ mol^2^/m^6^	(25)
ρ_p_	2210 kg/m^3^	(26)
*M*_p_	128 g/mol	

The Monte Carlo simulations have been executed on 10,000 parameter sets chosen randomly on the domain shown in Table 2. The goodness-of-fit is quantified with reduced chi-squared statistics
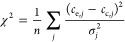
22from the corresponding measured (*c*_e,*j*_) and calculated (*c*_c,*j*_) values, where σ_*j*_ is the deviation of the experimental concentration at the *j*th sample point, and *n* is the degree of freedom. The experimental measurements are accessible up to 300 s; therefore, the numerical simulations are performed up to the same time range for each initial concentration. The six experimental data sets are sampled at every 0.5 s starting from 0, therefore overall *m* = 3606 points are used for the fitting, providing *n* = 3603 for the degree of freedom. The best results of the Monte Carlo simulation are then further optimized with a simplex algorithm.

**Table 2:** Parameter Domains Used for Monte Carlo Simulations

quantity	domain
log_10_(*k*_n_/[m^3^ mol^–1^ s^–1^])	(−10)–(+10)
log_10_(*k*_g_/[m^4^ mol^–1^ s^–1^])	(−10)–(+10)
*B*	(50)–(300)

## Results and Discussion

**Figure 3:**
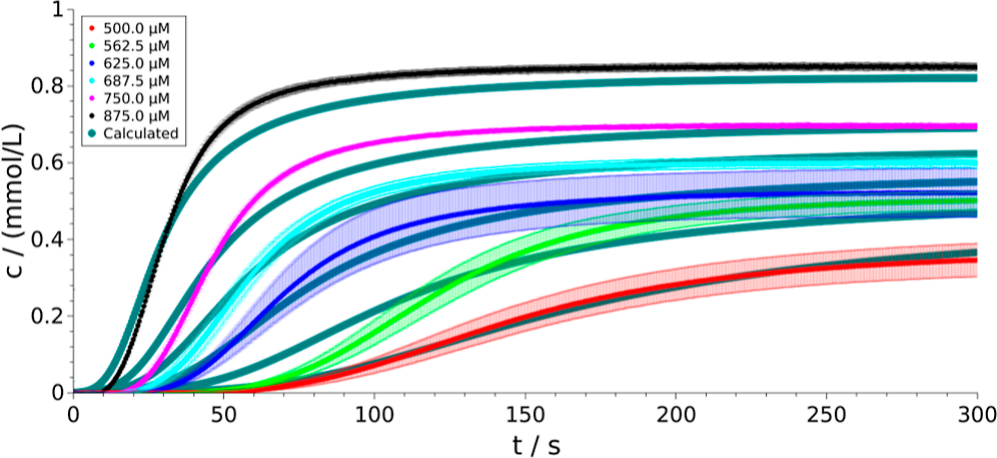
Temporal change of measured (colored symbols) and calculated (green solid lines) product concentrations at various initial reactant concentrations.

The optimized parameter set with a minimum of χ^2^ = 15.24 has the rate coefficient of *k*_n_ = (6.0 ± 1.0) × 10^–5^ m^3^ mol^–1^ s^–1^, the rate coefficient of *k*_g_ = (3.7 ± 1.0) × 10^–3^ m^4^ mol^–1^ s^–1^, and the thermodynamic parameter of *B* = 158.5 ± 0.1. Using these constants, the calculated concentration evolutions are compared to the experimental runs in Figure 3. In order to provide a physical representation of the goodness-of-fit, the average concentration deviation  is determined with the formula of
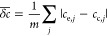
23

Using the calibration curve obtained from Figure 2, it is then converted to average turbidity deviation , which is within the experimental precision of turbidity measurements. To have a deeper insight into what influences the goodness-of-fit, we have calculated  separately for each concentration. They fall in the range of (2.2–13.3) × 10^–3^ with the largest uncertainties associated with the measurements of *c*_init_ = 562.5 μm (light green symbols in Figure 3) and 875 μm (black symbols in Figure 3). In the former, the greater deviation may originate from the experimental errors, while in the latter, it originates from the conversion of turbidity values to concentrations by using the calibration curve (Figure 2), which overestimates the equilibrium concentration at high initial concentrations.

The calculations return the main characteristics of the temporal evolution of the formed product (Figure 3) regardless of the initial concentration: they always show an early stage, where the amount of precipitate barely increases, followed by a rapid rise that later leads to saturation. The induction time (*t*_ind_) is a measure of the transition between the two regimes. It is a function of supersaturation according to
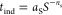
24where *a*_S_ and *n*_S_ are phenomenological parameters.^24^ For the simulation, the induction time is obtained from the intercept of the straight lines fitted to the calculated data points in the first and second regimes. There is a small difference in the estimated induction times: 21% in the exponent (*n*_S_) and 4% in the parameter *a*_S_ of eq 24, as shown in Table 3. The deviation may arise from the experimental method of acquiring induction times, since the intercept of the two fitted lines falls in a region where the turbidity is small; therefore, the deviation of the data is high.

**Table 3:** Experimental and Calculated Parameters of the Phenomenological Eq 24

parameter	experiment	calculation
*a*_S_	6.31 ± 0.48	6.03 ± 0.24
*n*_S_	2.91 ± 0.20	3.51 ± 0.06

**Figure 4:**
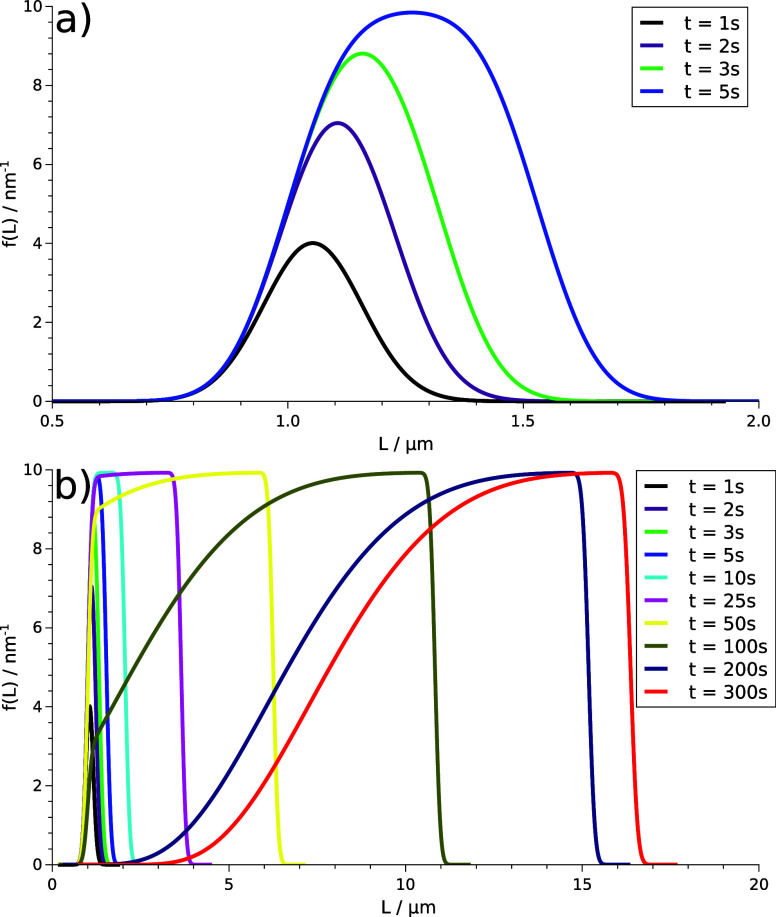
Evolution of PSD for *c*_init_ = 500 μm (a) in the first few seconds and (b) during the entire precipitation process.

The temporal evolution of the PSD function is presented in Figure 4. At the beginning of the precipitation process, the bell shape is sustained (Figure 4a), therefore in the initial few seconds, nucleation is dominant, corresponding to the initial part with a small slope for the selected case of *c*_init_ = 500 μm in Figure 3. It is followed by a regime where the PSD function starts to widen and the nucleation and growth processes become comparable. Beyond that, particle growth dominates: small particles no longer nucleate; only the existing ones grow. Thus, a shoulder will form on the left side of the *f*(*L*) curve, which is associated with the greatest slope in Figure 3. From *t* = 200 s, the distribution curve barely changes because the saturation region is reached, in which the reactants are depleted.

**Figure 5:**
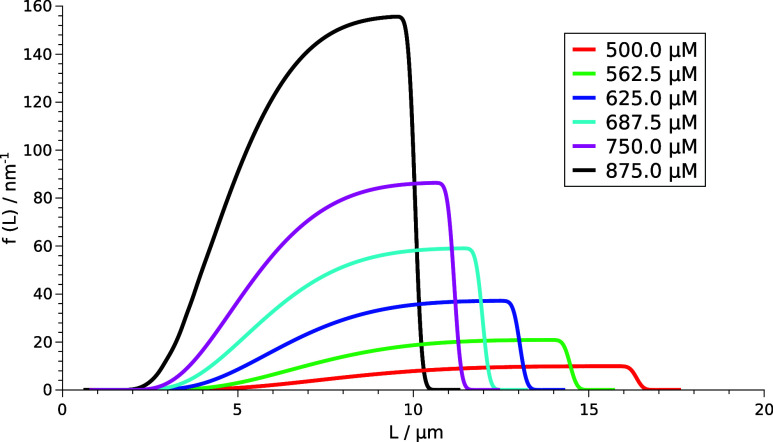
PSD at *t* = 300 s with different initial concentrations.

The final PSD depends on the initial concentrations, according to Figure 5. At higher initial concentrations, the number of particles is larger, as indicated by the greater area under *f*(*L*), however, they tend to be smaller in size. With decreasing supersaturation, the distribution curve flattens and widens toward the larger diameter sizes with decreasing area under *f*(*L*), i.e., resulting in fewer but bigger particles. This is in agreement with the experiments: at high supersaturation, nucleation is dominant, while at low, particle growth is dominant.

From the SEM image reported,^15^ the smallest and largest particles are measured with ImageJ software and their diameters are determined to be *D* = 1.84 μm and *D* = 8.25 μm. There is not sufficient data to create a statistical comparison, but it is adequate to conclude that the predicted PSD is in the same order of magnitude as in the experiments.

As an additional result of the fitting, the thermodynamic parameter *B* is also determined, from which the specific interfacial energy γ of calcium oxalate monohydrate crystals is calculated by substituting the constants in Table 1 into eq 12, yielding γ_calc_ = 38.4 mJ/m^2^. The experimentally determined specific interfacial energy for calcium oxalate monohydrate is γ_exp_ = 32.4 mJ/m^2^.^27^ The good agreement validates our method to provide predictions for the specific interfacial energy, as well.

## Conclusions

A model has been developed for precipitation processes to predict the dynamics of PSD by using product concentration time dependencies. Due to the high number of particles, population mass balance theory is used to describe the heterogeneous system. Unlike in many previous works utilizing a DPB model, experimentally measured temporal product concentration curves are used for parameter optimization instead of experimental PSDs. With this method, the evolution and the final size distributions of precipitate particles can be obtained; moreover, the specific interfacial energy can be estimated.

We have shown that the calculated concentration evolutions match the experimental curves within the range of the experimental errors. The temporal dynamics of PSDs at various initial concentrations reveals that with decreasing supersaturation, the distribution curves tend to flatten and shift toward the higher particle sizes. At high initial concentrations, nucleation is dominant, resulting in smaller but more particles. The bell shape of the distribution curve originating from the Gauss probability function is not sustained because the initial nucleation-governed regime is followed by a growth-dominated one, where significant particle growth accompanies nucleation. With comparable growth and nucleation rates, the distortion of the normal distribution shape occurs at the left side of the PSD but leaves the sharp cut-off at the right side of the curve.

The calculated specific interfacial energy value and particle sizes are compared to experimental ones, and they are also in good agreement. Hence, this approach is an appropriate method for precipitation reactions to predict the PSD and specific interfacial energy values from turbidity measurements.

Our model is applied to a system of high supersaturation by assuming isotropic precipitate particles. A systematic extension of our proof-of-concept study is the inclusion of particle geometry. This can be achieved by modifying the equations describing the volume and surface area of the particles. The numerical treatment can also be further extended to account for the dissolution of particles, and with size-dependent solubility, it can model Ostwald ripening.
